# The psychological process and support needs of HIV-positive individuals who disclose their status to sexual partners: a qualitative study

**DOI:** 10.3389/fpubh.2026.1870907

**Published:** 2026-06-16

**Authors:** Chen Pan, Caifeng Luo, Aixian Li

**Affiliations:** 1 School of Medicine, Jiangsu University, Zhenjiang, Jiangsu, China; 2 The Third People’s Hospital of Changzhou City, Changzhou, Jiangsu, China; 3 The Kunshan Women and Children’s Healthcare Hospital, Kunshan, Jiangsu, China; 4 The Affiliated Kunshan Hospital of Jiangsu University, Kunshan, Jiangsu, China

**Keywords:** disclosure preference, HIV, qualitative research, sexual partner disclosure, thematic analysis

## Abstract

**Objective:**

To explore the psychological journey and core needs of People living with HIV (PLWH) intending to disclose their status to sexual partners, providing a theoretical basis for constructing a disclosure support system.

**Methods:**

A descriptive qualitative study was conducted using semi-structured interviews with 15 People living with HIV at a hospital in Changzhou, China. Thematic analysis was used to analyze the interview data.

**Results:**

Five themes were identified regarding disclosure to sexual partners: (1) disclosure motivation and internal drivers; (2) risk perception during the disclosure process; (3) preparation and perceived capacity for disclosure; (4) safety protection strategies; and (5) external and social support needs. These themes reveal a multidimensional and interwoven risk perception system in disclosure decision-making, including fears of discrimination, relationship breakdown, social stigma, and threats to economic security. They further systematically outline the proactive and strategic safety behaviors individuals employ to mitigate such risks, including scenario selection, information control, and exit planning.

**Conclusion:**

The findings highlight that the decisions of People living with HIV to disclose their status to sexual partners involve a dynamic interplay of emotional, ethical, and stigma-related pressures. Clinical care should establish dedicated disclosure counseling roles, provide role-play-based communication skills training, and build multidisciplinary support networks.

## 1 Introduction

Human Immunodeficiency Virus (HIV) remains a significant public health challenge. Globally, an estimated 40.8 million people were living with HIV at the end of 2024, with approximately 1.3 million new infections occurring that year (UNAIDS, 2025) (1). In China, the situation is similarly critical, with around 1.387 million PLWH as of June 2025 (2). Sexual transmission remains the predominant mode of HIV infection. Among new diagnoses in China, heterosexual transmission accounted for 73.0% (34,368 cases) and male-to-male sexual transmission accounted for 24.8% (11,653 cases) (2).

Partner notification refers to the public health practice of contacting past sexual partners of newly diagnosed individuals to offer HIV testing. This strategy aims to reduce high-risk sexual behaviors among partners and to promote HIV counseling and testing (3). It is important to distinguish between two related but distinct constructs. Disclosure, in contrast, refers to the voluntary sharing of one’s HIV status with a current or prospective sexual partner. This study focuses on the latter—disclosure to current or potential sexual partners. From both clinical and public health perspectives, disclosure is therefore considered a vital step in interrupting HIV transmission and protecting partners’ right to health, while also aligning with legal and ethical obligations for risk reduction (4).

Importantly, all participants in this study were virally suppressed, meaning they had achieved undetectable viral loads. According to the Undetectable = Untransmittable (U=U) consensus established by multiple large-scale studies, individuals with undetectable viral loads do not sexually transmit HIV to their partners (5). This biomedical reality fundamentally reshapes the ethical and psychological calculus of disclosure: Under such a consensus, researchers should no longer assume by default that “non-disclosure = irresponsible” or “non-disclosure = a potential threat to a partner”. On the contrary, it should be recognized that for viral suppressors, disclosure decisions are mainly influenced by the following factors rather than medical necessity: fear of the partner’s reaction, assessment of relationship stability, past experiences of discrimination, and the degree of internalized social stigma. For virally suppressed individuals, disclosure becomes primarily a matter of relational trust and stigma navigation rather than transmission prevention (6). Therefore, the focus of qualitative interview analysis should shift from “whether the disclosure obligation has been fulfilled” to “the meaning and cost of disclosure or non-disclosure in the context of the individual’s life”—for example, non-disclosure may be a self-protection strategy rather than a sign of disrespect to a partner. Understanding disclosure through this lens is essential to avoid inadvertently stigmatizing PLWH who choose not to disclose.

Research indicates that disclosing one’s HIV status to family and friends is associated with enhanced social support, improved long-term treatment adherence, better antiretroviral therapy compliance, and reduced psychological distress, all of which serve as protective factors for sustained engagement in care (7, 8). Nevertheless, a significant intention-behavior gap persists. Studies indicate that only about 65% of PLWH disclose their status to sexual partners, and such disclosures are often inconsistent or delayed (9). Research in the Chinese context shows that the disclosure rate of HIV infection to spouses among PLWH ranges from approximately 56% to nearly 80%, yet a considerable proportion still choose to conceal their infection status from their spouses (10). Key barriers include stigma, fear of discrimination, social exclusion, and financial pressures (11–13).

To address these challenges, various intervention strategies have been proposed. For instance, interventions designed to support parents living with HIV in disclosing their status to children have been shown to enhance self-efficacy and reduce anxiety (14). Additionally, medical assistance strategies in where healthcare providers facilitate disclosure, have proven effective in promoting disclosure and encourages sexual partners to undergo HIV testing (15). Furthermore, behaviorally informed interventions—such as youth-friendly visual tools (e.g., invitation cards, animated videos), SMS notifications, and expedited partner treatment, have been proposed to alleviate the psychological distress associated with disclosure (16, 17).

However, the applicability of these strategies within the Chinese context remains limited. Furthermore, previous research has focused on individuals who intend to disclose their HIV status but have not yet done so. This study aims to systematically explore the psychological journey, core dilemmas, and support needs of PLWH who intend to disclose. Rather than placing the entire burden of HIV-related communication on PLWH, we adopt a relational framework that acknowledges shared responsibility within sexual partnerships (18). The findings are intended to inform interventions that support both partners in these conversations, not PLWH alone.

## 2 Materials and methods

### 2.1 Study design

This study employed a descriptive qualitative design to gain an in-depth understanding of the experiences, strategies, and support needs of PLWH in the process of preparing for or undertaking disclosure to their sexual partners.

### 2.2 Participant selection

A purposeful sampling strategy was used to recruit individuals living with HIV from the HIV inpatient ward or outpatient clinic of a hospital in Changzhou, a prefecture-level city located in the developed Yangtze River Delta region in southern Jiangsu Province, China (i.e., an urban area), between October 2025 and January 2026. Participants were identified and selected through the following procedure: First, trained clinical nurses screened all patients presenting for routine care or admission based on the study’s inclusion and exclusion criteria. Eligible patients were then briefly introduced to the study by the nurses. If a patient expressed initial willingness, a research assistant (not involved in clinical care) provided detailed information, reconfirmed eligibility via a short structured interview, and invited voluntary participation. A maximum variation purposive sampling approach was applied to capture diversity in age, gender, time since diagnosis, and relationship duration. All participants provided written informed consent prior to enrollment.

Inclusion criteria: (1) aged 18–60 years (this age range was selected to focus on adults in their active working and intimate relationship years, as older adults face different disclosure-related challenges beyond this study’s scope); (2) diagnosed with HIV infection and receiving antiretroviral therapy for at least 6 months (this duration ensures that participants have achieved sufficient clinical stability, psychological adaptation to their diagnosis, and familiarity with the U=U concept, all of which are relevant to their disclosure decision-making process); (3) having a stable sexual partner (not all participants were currently sexually active, but all had a stable partner); (4) expressed willingness or intention to disclose their status but had not yet done so, or reported hesitation in disclosure; (5) able to articulate personal experiences and feelings clearly and voluntarily participate in in-depth interviews; (6) provided informed consent.

Exclusion criteria: (1) HIV infection via non-sexual routes (e.g., mother-to-child transmission or blood exposure)—excluded because this study focuses specifically on disclosure dynamics related to sexually acquired HIV within stable sexual partnerships; (2) preliminary violence risk assessment indicating high risk; (3) partner who were already aware of the participant’s HIV status; (4) severe cognitive impairment or psychiatric disorders preventing effective communication; (5) recent major negative life events (e.g., death of a loved one, relationship breakdown) causing extreme emotional instability; (6) concurrent participation in other interventional research that could potentially confound the study findings.

### 2.3 Sampling and recruitment process

Initial participants were purposively selected for their potential to provide rich, relevant information, determined through a brief screening conversation assessing their ability to articulate disclosure-related thoughts and emotions. A maximum variation sampling strategy was intentionally used to ensure diversity in gender, age, marital status, and sexual transmission route. Following preliminary analysis of early interviews, subsequent participants were recruited to test, refine, and expand upon the emerging themes. Recruitment continued until thematic saturation was achieved, defined as the point at which three consecutive interviews yielded no new themes or subthemes. This resulted in a final sample of 15 participants.

### 2.4 Data collection

#### 2.4.1 Developing the interview guide

Semi-structured in-depth interviews were conducted. The interview guide was initially developed based on the Information-Motivation-Behavior model (19) and a review of relevant literature. Specifically, the three IMB components guided the five interview domains: Information (domains 1 and 2 on understanding, motivations, and risk assessment); Motivation (domains 1 and 4 on disclosure drivers and anticipated partner reactions); and Behavioral Skills (domains 3 and 5 on communication strategies and support needs). After conducting two pilot interviews (data not included in the final analysis), the wording of questions was refined to form the final version (Supplementary data 1). The interview content encompassed the following five domains:

(1) Participants’ understanding of and motivations for disclosure decisions;
(2) Risk assessment and concerns during the disclosure process;
(3) Preparatory actions and planned communication strategies before disclosure;
(4) Potential partner reactions and anticipated responses;
(5) Needs for practical support and professional assistance.

#### 2.4.2 Interviews procedure

A preliminary violence risk assessment was performed prior to the interviews to screen for potential harm to participants or researchers. This assessment was adapted from the Chinese Risk Assessment Tool for Victims (CRAT-V) (20), a validated IPV risk tool for Chinese populations. We selected only the CRAT-V items relevant to HIV disclosure-related violence: (1) history of intimate partner violence; (2) current relationship conflict; (3) partner’s substance use; and (4) partner’s history of aggressive behavior - and excluded other CRAT-V items (e.g., social isolation) that were not directly pertinent to the disclosure context. This assessment was included because prior research indicates that disclosure can trigger intimate partner violence. If a high risk was identified, the interview was postponed, and established clinical safety protocols were initiated, such as contacting the participant’s primary physician or mental health counselor. Interviews were conducted by two female registered nurses with over 5 years of experience in HIV clinical care. Both held a Bachelor’s degree in Nursing and had received prior training in qualitative research methods, including a 16-h workshop on sensitive-topic interviewing. They completed an additional 8-h study-specific training session covering the interview guide, ethics, and non-directive probing techniques. Neither nurse was directly involved in participant’s routine clinical care (e.g., medication administration or treatment decisions), although they worked in the same clinical setting and had occasional professional contact with some participants. To minimize any influence of this prior relationship, a research assistant (not the nurse-interviewers) initially approached potential participants and obtained informed consent; participants were explicitly assured that declining or withdrawing would not affect their medical care; interviewers adopted a non-judgmental, empathetic stance; and interviews were conducted in private rooms outside the inpatient ward. A physician was present during each interview to assess for any risk or emotional distress. Interviews took place in private, quiet, and secluded rooms within the hospital. All interviews were audio-recorded; immediately after each session, the recording was de-identified using a unique participant code and stored on a password-protected, encrypted hospital computer. Paper documents (consent forms) were kept in a locked filing cabinet in a locked research office, accessible only to the principal investigator. All research team members signed confidentiality agreements. Informed consent forms were signed before the interview, during which the study objectives and confidentiality principles were explained in detail, including statements that data would be anonymized, that HIV status and disclosure information would remain confidential, and that no personal identifiers would appear in any publication. No interviews were suspended due to emotional distress in this study. Had severe emotional distress occurred, the researcher would have suspended the session and offered psychological support, with professional counseling arranged if deemed necessary. Each interview lasted approximately 30–60 min.

#### 2.4.3 Data management

All interview recordings were transcribed verbatim by two researchers within 24 h and subsequently de-identified. Transcripts were returned to the respective participants for verification to ensure accuracy and completeness of the data. Verification was conducted as follows: each participant received a printed copy of their de-identified transcript in a sealed envelope within 1 week post-interview. Participants were asked to read the transcript, mark any inaccuracies, add any missing information, or remove any content they wished. They had up to 7 days to return the transcript in a sealed envelope or via secure encrypted email. Of the 15 participants, 12 confirmed accuracy without changes; three made minor clarifications (e.g., rewording for clarity, adding a few words). No participant requested removal of any content. All corrections were reviewed by two researchers and incorporated into the final analysis only when agreed upon as clarifications rather than post-interview new information.

### 2.5 Data analysis

Data management was executed using NVivo version 11.0, employing Braun and Clarke’s thematic analysis method for analysis (21). We followed their six-phase framework (familiarization, coding, theme searching, theme reviewing, theme defining and naming, and report writing). The analysis explicitly distinguished core themes (overarching concepts) from subthemes (specific dimensions within each core theme). To maintain transparency and rigor, two researchers independently performed initial coding and theme development, meticulously documenting the coding process and rationale for theme generation. To ensure descriptive and interpretive validity, the researchers collaboratively reviewed the interview materials with the participants.

## 3 Results

### 3.1 Demographic characteristics of participants

This study involved 15 PLWH who were willing to disclose their status to sexual partners, with a mean age of 32 years (range: 18–50 years). The majority were employed (*n* = 12, 80.0%), unmarried (*n* = 8, 53.3%), and possessed a college degree or higher (*n* = 7, 46.7%). Five participants reported monthly incomes exceeding ¥5,000. Detailed demographic data are presented in Table 1.

**Table 1:** Demographic characteristics of participants (*N* = 15).

Characteristic		Number of participants N (%)
Gender	Female	8(53.3)
	Male	7(46.7)
Age (years)	18–25	3(20)
	26–35	6(40)
	36–45	5(33.3)
	45–60	1(6.7)
Occupational category	Student/unemployed	4(26.7)
	Office/professional/technical	5(33.3)
	Service industry	3(20.0)
	Self-employed	2(13.3)
	Worker on sick leave	1(6.7)
Monthly income	<3,000	2(13.3)
	3,000–5,000	5(33.3)
	>5,000	5(33.3)
	No income	3(20.0)
Education	High school or below	6(40.0)
	College diploma	2(13.3)
	Bachelor’s degree or above	7(46.7)
Marital status unmarried	Unmarried	8(53.3)
	Married	3(20.0)
	Divorced	4(26.7)
HIV transmission route	Male-to-male sexual contact	6(40.0)
	Heterosexual transmission	5(33.3)
	Commercial sex work	2(13.3)
	Unknown/other	2(13.3)

### 3.2 Core themes of the informing dilemma

Through repeated reading and analysis of the interview data, five core themes and 18 sub-themes were identified in Table 2.

**Table 2:** Core themes and subthemes of PLWH’s experiences in disclosing their status to sexual partners.

Core theme	Subtheme	Number of respondents (*N* = 15)
1. Disclosure motivation and internal drivers	Motivation of love and responsibility	11
	Psychological burden relief	8
	Principles of moral integrity	7
	Legal and ethical constraints	4
2. Risk perception during the disclosure process	Fear of discrimination	13
	Fear of information leakage	9
	Economic and survival pressure	6
	Family breakdown and career loss	8
	Impact of social stigma	10
3. Preparation and perceived capacity for disclosure	Information preparation	12
	Self-psychological preparation	11
	Interpersonal practice and communication skills	10
	Choice of timing and location for disclosure	9
	Communication strategies During disclosure	11
4. Safety protection strategies	Proactive defense Before disclosure	7
	Defensive measures during disclosure	9
5. External and social support needs	External professional support	15
	Social support	8

Numbers indicate the count of participants who mentioned each subtheme at least once during the interview. The total number of participants is 15.

#### 3.2.1 Disclosure motivation and internal drivers

##### 3.2.1.1 Motivation of love and responsibility

Most respondents viewed “love” as the core driving force behind disclosure, believing it to be an act of respect and responsibility toward their partner.

*P12*: “We can plan together for a healthy future.”

*P10*: “I hope he chooses to continue our relationship because he values our bond.”

*P08*: “If he shows hesitation, I’d say, ‘I understand you need time to process this, and I respect whatever decision you make.’ I won’t beg.”

These narratives reveal that love functions not merely as an emotional bond but as a moral justification for disclosure. Unlike previous studies that framed disclosure primarily as a risk management strategy (3), our participants constructed disclosure as an act of intimacy-building and shared future planning. P12’s reference to “planning together for a healthy future” illustrates how disclosure was perceived as a gateway to shared life planning rather than merely a risk to be managed. P10’s hope that his partner “chooses to continue the relationship because he values our bond” reveals a desire for acceptance based on the totality of the relationship, not sympathy or obligation. This finding tentatively suggests that for the participants in this study, disclosure may be embedded within a relational ethic of mutual care, where love legitimizes the disclosure act and potentially buffers against anticipated rejection.

##### 3.2.1.2 Psychological burden relief

Some infected individuals experience intense guilt and anxiety from long-term concealment of their condition, urgently seeking psychological relief through disclosure.

*P01*: “Every time I fail to take proper precautions, I feel terribly guilty toward him—my conscience just won’t let me rest.”

*P12*: “This protects him and frees my own conscience.”

These statements reveal an additional function of disclosure—self-redemption. Notably, P01 specifically mentioned “failure to take proper precautions,” indicating that guilt arises not from HIV infection itself but from the perceived failure to adequately protect one’s partner during intimate encounters. This finding aligns with prior research demonstrating that concealment imposes significant psychological costs (8). The contribution of this study is to reveal that the drive to disclose stems not only from responsibility toward others but also from the need to maintain one’s moral self-integrity—restoring a damaged self-image and reclaiming an identity as “a good person” through disclosure. This dual motivation (altruistic and self-oriented) has important implications for intervention design: disclosure support programs may benefit from emphasizing both the protective value for partners and the mental health benefits for the disclosing individual.

##### 3.2.1.3 Principles of moral integrity

Integrity and morality emerged as key reasons cited by some interviewees for insisting on disclosure.

*P13*: “I have an obligation to let him know so he can choose whether to take the risk.”

*P15*: “If I don’t tell him, I’d be a liar and a bad person.”

These statements reflect participants’ commitment to the “moral self.” Notably, P15 described herself in absolute, binary moral terms—“a liar and a bad person”—suggesting that non-disclosure is internalized not merely as a behavioral choice but as an identity threat. This finding extends existing literature: prior research has primarily focused on external barriers to disclosure (e.g., stigma, discrimination), whereas this study reveals how internalized moral pressure drives disclosure independently of external constraints. Even in the absence of external mandates, participants chose to disclose due to internalized moral standards.

##### 3.2.1.4 Legal and ethical constraints

Some interviewees referenced legal provisions regarding the duty to disclose serious illnesses, asserting that disclosure is not only a moral choice but also a legal obligation.

*P14*: “The Civil Code also stipulates that concealing serious illnesses before marriage can lead to annulment.”

*P13*: “I proactively researched relevant legal precedents and ethical guidelines.”

This subtheme has rarely been reported in prior literature and may reflect increasing legal awareness in the Chinese context. Participants’ proactive efforts to consult legal texts (as described by P13) suggest that disclosure decisions have moved beyond the personal-emotional realm into rational consideration of social rules and institutional constraints. Notably, both participants who cited legal provisions (P13, P14) were highly educated young men, suggesting that future research should examine the impact of educational level and legal awareness on disclosure decisions. Furthermore, legal provisions appear to serve as a “justification tool”—both for self-persuasion and as potential talking points during disclosure conversations with partners.

#### 3.2.2 Risk perception during the disclosure process

##### 3.2.2.1 Fear of discrimination

“Fear of discrimination” in this study captures expectations of external negative actions (e.g., social death, silent treatment) rather than internalized stigma, which is coded under “impact of social stigma.” The fear of HIV-related stigma was the most common psychological barrier among interviewees.

*P11*: “I’m afraid she’ll tell others, ‘Look, he’s just a useless person.”

*P15*: “What I dread most is social death at school.”

*P08*: “But I’m afraid he won’t be able to accept it psychologically and will use silent treatment to force us to break up. That kind of slow torture is even more painful.”

These statements reveal three levels of fear. The first is depreciation of social identity (P11: “a useless person”), reflecting fear of being socially labeled as a failure. The second is exclusion from social networks (P15: “social death”), a term commonly used by younger participants that reveals extreme sensitivity to social circle rejection. The third is chronic relationship erosion (P08: “silent treatment... slow torture”). Unlike prior research focusing on acute negative reactions (e.g., breakup, violence), P08 identified a more insidious but equally painful fear—not outright rejection, but sustained emotional withdrawal. This finding has important intervention implications: post-disclosure support should address not only crisis intervention but also relationship repair and emotional adjustment.

##### 3.2.2.2 Fear of information leakage

The fear of privacy breaches has made some interviewees highly cautious about disclosure.

*P14*: “My biggest worry? Not that he’ll look down on me, but that he’ll go around telling everyone.”

*P15*: “The worst outcome would be him blocking me and posting in the class group chat: ‘XX has HIV, everyone be careful.’”

These statements shift the focus from the partner’s reaction to broader social exposure. P14’s distinction between “looking down on me” (personal rejection) and “telling everyone” (social exposure) is particularly instructive—it suggests that for some participants, loss of control over personal information is more threatening than the partner’s individual response. P15’s worst-case scenario involves complete loss of reputational control through social media, reflecting how digital environments have created new dimensions of disclosure risk not present in earlier research.

##### 3.2.2.3 Economic and survival pressures

Some respondents feared that disclosure could trigger a chain reaction leading to unemployment, business damage, or disruption of income sources.

*P11*: “All I have now is a pile of U=U materials and an unemployment certificate.”

*P08*: “If word gets out, my entire business could be affected.”

These statements reveal that disclosure risks extend beyond the personal and relational domains into economic survival. P11’s juxtaposition of “U=U materials” (scientific evidence of zero transmission risk) with “unemployment certificate” (evidence of economic precarity) poignantly illustrates the gap between biomedical facts and social realities. Despite being virally suppressed and posing no transmission risk, participants remained vulnerable to economic consequences of disclosure—a finding that underscores the limits of U=U in addressing structural forms of stigma.

##### 3.2.2.4 Family breakdown and career loss

Married or divorced respondents commonly feared that disclosure would trigger family conflicts, loss of child custody, or damage to their professional reputation.

*P02*: “I’m more afraid he’ll divorce me, take the kids away, and leave us mother and child homeless.”

*P13*: “In the worst-case scenario, if it causes a dispute, how much damage would it inflict on my career?”

These statements reflect concerns about life-stability threats that could follow disclosure. For P02, a married woman with a child, the feared consequences extend beyond herself to her child’s welfare and housing stability. For P13, career damage represents a threat to long-term life trajectory. Although family breakdown and career loss are qualitatively different consequences, both represent threats to fundamental life stability—distinguishing them from the interpersonal rejection fears expressed by younger, unmarried participants.

##### 3.2.2.5 Impact of social stigma

This subtheme captures how participants experience and internalize social stigma, as distinct from their fear of encountering future discriminatory behaviors. Some interviewees indicated that social prejudice and institutional exclusion profoundly affect the psychology and behavior of infected individuals.

*P02*: “I feel like a criminal right now—I’m afraid that if I don’t tell the other person, I could transmit HIV to them; but if I do tell them, they will look down on me with contempt.”

*P07*: “Even in my industry, where HIV awareness is relatively advanced, the stigma persists.”

P02’s self-characterization as “a criminal” reflects profound internalized stigma absorbed from social and legal discourses surrounding HIV. Importantly, this self-perception persists despite her being virally suppressed and posing no transmission risk—illustrating how stigma operates independently of biomedical reality. P07’s observation that stigma persists even in industries with “relatively advanced HIV awareness” suggests that knowledge alone is insufficient to eliminate stigma, pointing to the need for interventions targeting attitudes and behaviors, not just information.

#### 3.2.3 Preparation and perceived capacity for disclosure

##### 3.2.3.1 Information preparation

Most respondents gathered medical materials through professional platforms (such as CDC, Up To Date), organized test results and U=U information to enhance credibility during disclosure.

*P12*: “I prepared all test results from the past six months and organized them into a folder.”

*P15*: “I repeatedly reviewed materials from professional sources like Up To Date and CDC guidelines.”

These statements reveal that participants actively prepared evidentiary materials to support disclosure. The act of organizing medical records into a “folder” suggests a strategic approach—participants anticipated that partners might doubt their claims and prepared scientific evidence in advance. This finding challenges deficit-based narratives that portray PLWH as passive or helpless in disclosure situations, revealing instead considerable agency and strategic thinking.

##### 3.2.3.2 Self-psychological preparation

Some interviewees indicated that prior psychological preparation is necessary before disclosure, involving managing feelings of self-blame and shame to enhance confidence and improve disclosure success rates.

*P13*: “I would seek psychological counseling or support from Red Ribbon Home to address these emotions of self-blame and shame.”

*P10*: “To increase the likelihood of a positive outcome, I must establish an image of extreme reliability and rationality in his presence, convincing him of my judgment and control capabilities.”

P10’s statement is particularly revealing—she recognized that successful disclosure depends not only on what she says but on how she is perceived. By emphasizing “reliability and rationality,” she attempted to preempt potential concerns about her judgment and self-control. This suggests that disclosure is not merely a factual transmission but a performance of credibility, where the disclosing individual must simultaneously manage emotional vulnerability and project competence.

##### 3.2.3.3 Interpersonal practice and communication skills

Respondents consistently called for professional organizations to provide “pre-disclosure training,” including role-playing, communication techniques, and script rehearsals.

*P09*: “The most practical thing would be to train me before the disclosure, telling me exactly what to say.”

*P07*: “Before the disclosure, I desperately need role-playing and feedback tailored to my specific situation.”

These requests reveal a critical gap in existing support services. Participants did not merely want general counseling or emotional support—they wanted concrete, practical, scenario-based communication training. The call for “role-playing” and “feedback tailored to my specific situation” suggests that generic advice is insufficient; participants needed personalized rehearsal opportunities.

##### 3.2.3.4 Choice of timing and location for disclosure

Respondents tend to choose non-domestic settings and emotionally stable periods for disclosure to minimize conflict risks.

*P02*: “I would prioritize weekends after dinner, when there’s no work pressure.”

*P09*: “I’d choose a weekend daytime when my child is at their extracurricular class, and arrange to talk with them on a less crowded park bench.”

These statements reveal strategic environmental planning. The preference for non-domestic settings (P09’s “park bench”) and times when children are absent suggests that participants sought to control environmental variables to reduce potential volatility. The selection of emotionally stable periods (P02’s “no work pressure”) indicates an understanding that disclosure success depends partly on the partner’s emotional state, not just the content of the message.

##### 3.2.3.5 Communication strategies during disclosure

Respondents consistently emphasized using “love” and ‘sincerity’ as opening strategies, with some also preparing apologies, phased disclosure, and emphasizing family protection measures.

*P12*: “I would take his hand and say, ‘I love you, and precisely because of that, there’s something about my health I need to tell you.’”

*P14*: “He yelled at me, and I just kept apologizing, saying ‘I’m sorry, I’m sorry, I didn’t mean to deceive you.’”

*P08*: “I would shave alone at home, and if I got a cut, I’d cover it with a waterproof bandage.”

These statements reveal diverse communication strategies. P12’s strategy frames disclosure within an affirmation of love—starting with reassurance before delivering difficult news. P14’s response to a negative reaction—repeated apology—suggests that even well-prepared participants may default to appeasement when faced with partner anger. P08’s mention of shaving and waterproof bandages reveals attention to even minor details that might signal risk (e.g., a shaving cut that could bleed), demonstrating hypervigilance about any cue that might trigger partner anxiety.

#### 3.2.4 Security protection strategy

##### 3.2.4.1 Proactive defense before disclosure

Some interviewees enhance their sense of security before disclosure through legal measures, information concealment, and highly cautious disclosure decisions.

*P13*: “I will never tell them which specific building I live in—I’ll only reveal the neighborhood information, since we always go out on dates.”

*P08*: “I have to be extremely cautious.”

These statements reveal a level of precaution that extends beyond typical disclosure preparation. P13’s decision to reveal only the neighborhood, not the specific building, suggests that some participants maintained protective barriers even with partners they intended to inform. This finding extends existing literature by identifying “risk-averse preparation” as a distinct dimension of disclosure behavior—actions taken not in response to actual threats but in anticipation of potential negative reactions.

##### 3.2.4.2 Defensive measures during disclosure

Respondents commonly choose public places for disclosure and prepare “escape tools” such as electric bikes and temporary lodging.

*P06*: “I ride an electric bike—he can’t catch up with me.”

*P08*: “If things get too tense, I’ll immediately switch gears: I’ll say ‘Alright, got it. Bye,’ then pay and leave. No need for soothing words—everyone’s time is valuable.”

*P09*: “I choose public places so even if he gets angry, he won’t feel comfortable raising his voice.”

These statements reveal participants’ explicit planning for potential partner aggression. P06’s electric bike functions as an escape vehicle—an acknowledgment that physical safety may be threatened. P08’s pre-planned exit script (“pay and leave”) demonstrates that participants had rehearsed not only disclosure but also contingency plans. P09’s strategic use of public spaces as a violence deterrent (people “won’t feel comfortable raising his voice”) reveals sophisticated understanding of how social context constrains behavior. These findings suggest that disclosure support programs should address safety planning alongside communication skills.

#### 3.2.5 External and social support needs

##### 3.2.5.1 External professional support

Reflects the desire of PLWH for external assistance, such as from professional organizations, medical staff, psychologists, and peer support groups. Needs include legal aid, psychological counseling, peer companionship, practical life assistance, and educational support.

*P08*: “I wish an authoritative doctor could first call my husband to prepare him for the conversation.”

*P12*: “I need a psychologist who understands both HIV stigma and communication in intimate relationships.”

P08’s request for a doctor to “prepare” her husband before she discloses is particularly striking—it suggests that participants desired third-party mediation to manage partner reactions. This request implies that participants believed professional authority figures could achieve what participants themselves could not: convincing partners to respond constructively. P12’s request for a psychologist with dual expertise (HIV stigma and relationship communication) reveals that participants recognized the intersection of these domains—disclosure is not just a medical issue but a relational one requiring specialized support.

##### 3.2.5.2 Social support

Some respondents called for enhanced public awareness campaigns and legislative safeguards to reduce discrimination and stigma.

*P05*: “We hope society will help raise awareness about people like us to reduce discrimination.”

*P07*: “I really hope a protective law can be enacted for people like us.”

These statements shift responsibility from individual PLWH to structural actors. Unlike requests for personal counseling or skills training, these calls target public attitudes (P05) and legal frameworks (P07)—suggesting that participants recognized the limits of individual-level interventions. This finding indicates that while participants actively prepared and strategized, they also understood that full resolution of disclosure distress requires structural changes beyond individual control.

## 4 Discussion

This study employed in-depth interviews to systematically explore the intricate psychological journey, strategic preparations, and multifaceted support needs of PLWH who wish to disclose their status during the disclosure process. The findings not only validate existing literature that emphasizes the emotional, ethical, and social norms influencing disclosure behavior, consistent with previous findings (22), but also provide deeper insight into the fundamental contradictions, individual agency, and systemic support deficiencies faced in the shift from “desire to disclose” to “actual disclosure action.” The subsequent discussion integrates these findings with pertinent literature.

### 4.1 The disclosure decision: a multidimensionality and dynamic process

This study demonstrates that the decision-making process is not a binary choice but a dynamic psychological journey marked by internal conflict, as documented in a multi-country review (23). The motivations for disclosure are complex and interrelated, arising from perceptions of love and responsibility, moral integrity, legal obligations, and the desire to alleviate psychological burdens. Collectively, these factors serve as the primary impetus for disclosure a pattern also observed in prior studies (24, 25). Furthermore, positive social support received after disclosure can enhance the willingness to disclose and improve treatment adherence, as demonstrated in previous research (26). However, in contrast to studies that focus solely on the benefits of disclosure, our participants consistently reported a significant internal struggle between “driving forces” and “resistance.” For example, while some felt morally compelled to disclose (P02 feared “spreading it to them” if she did not tell), they simultaneously confronted substantial external threats: social stigma, fear of discrimination, potential relationship breakdowns, and economic survival pressures - barriers similarly identified in prior studies (27). Some participants notably expressed significant concerns regarding the potential for intimate partner violence following disclosure. Siemieniuk et al. similarly concluded that such disclosure may trigger intimate partner violence (28). To interpret this recurring pattern, we developed two analytical concepts: “the moral self” (the desire to be an honest, responsible partner) and the “survival self” (the fear for one’s own safety, relationships, and economic stability). These concepts are researcher-generated labels intended to summarize the internal conflict that participants described in their own words (e.g., P02’s “I feel like a criminal right now—afraid of both harming others and being looked down upon”; P08’s fear of “silent treatment…slow torture”; P15’s dread of “social death”; P11’s “pile of U=U materials and an unemployment certificate”). This recurring tension in participant’s narratives -between wanting to be an honest partner (the “moral self”) and fearing for one’s own safety and survival (the “survival self”)—is consistent with the dynamic described by self-regulation theory (29). Rather than claiming this conflict as a universal theoretical mechanism, we note that among our participants, this tension appeared to underlie the disparity between disclosure intention and actual behavior. This suggests that future interventions should aim to address this psychological tension. Rather than simply promoting disclosure, these interventions should assist infected individuals in recognizing and reconciling these internal conflicts as they are experienced in real-life situations.

### 4.2 Stigma and discrimination as core barriers to disclosure

Despite the increasing acceptance of the “U=U” (Undetectable = Untransmittable) concept (30), HIV-related stigma remains a major barrier to disclosure. Individuals fear not only partner rejection but also information leaks, damaged relationships and reputations, and family breakdown (31). Many internalize anticipated stigma, leading to self-stigma (32). For men who have sex with men, HIV intersects with minority identity, producing dual stigma that complicates disclosure (33), which complicates and intensifies disclosure situations. Brown et al.’s research highlights that social media frequently depicts PLWH as drug users, sex workers, and sexual deviants (34) and half of respondents in one study reported AIDS-related discrimination (35). Despite being virally suppressed, our participants remained deeply concerned about disclosure. As P11 noted, she had “a pile of U=U materials and an unemployment certificate”—scientific evidence of zero transmission risk did not eliminate fear of social and economic consequences. However, a fundamental tension exists between the scientific evidence of U=U and the socio-legal framing of disclosure as a moral or legal obligation. While U=U confirms zero transmission risk from virally suppressed individuals, many jurisdictions and ethical guidelines still require or expect HIV status disclosure to sexual partners regardless of viral load. This contradiction places PLWH in a double bind: following U=U logic and choosing not to disclose may invite legal prosecution or moral condemnation, while disclosing risks discrimination, relationship dissolution, and social death. Consequently, solely enhancing individual psychological resilience or teaching disclosure techniques is insufficient to fundamentally address the issue. Future interventions must target structural factors, such as strengthening anti-discrimination education and promoting rights-based legislation, to alleviate the source of “anticipated stigma” for individuals living with HIV.

### 4.3 Capacity building and defense strategies: manifestations of infected subjectivity

Our data show that PLWH are not passive regarding disclosure decisions. Participants in this study actively collected medical evidence (including U=U materials), strategically chose disclosure timing and locations, and rehearsed communication strategies in advance. For instance, several participants described practicing specific phrases to use during disclosure or choosing a semi-public venue for safety reasons. This pattern of active preparation challenges deficit-based narratives that portray PLWH as helpless in disclosure situations (36).

Notably, participants actively used U=U materials to support disclosure. Consistent with Jones et al. (37)^,^ who applied the U=U concept in disclosure interventions, our participants used U=U evidence not merely for their own reassurance but as a communication tool to reduce partners’ anxiety. One participant explicitly mentioned showing U=U information to a partner to explain “I cannot transmit the virus.” This finding suggests that when effectively communicated, U=U can function as a practical social resource that facilitates disclosure.

The theme of “safety protection strategies”—encompassing information concealment, venue selection, and evacuation planning—emerged directly from participant’s descriptions of how they prepared for potential negative reactions. Prior research has focused predominantly on either facilitating factors or barriers to disclosure (38). By staying close to what participants actually said they did, Our findings extend this binary framework by identifying a third dimension: risk-averse preparation. This offers a concrete basis, grounded in participant accounts, for developing disclosure support programs that address safety planning alongside communication skills.

### 4.4 Building a multidisciplinary collaborative support network

The support needs articulated by respondents highlight the existing fragmentation and systemic inadequacies within services. Research demonstrates that social support for PLWH/AIDS remains insufficient (39). While governments and healthcare institutions are pivotal in providing policy support, treatment, care, and health education—such as the “Four Frees and One Care” policy (40, 41)—our participants consistently requested more than routine psychological reassurance or information dissemination. Instead, there is a pressing need for systematic, personalized, and professional support throughout the entire disclosure process. Based on their accounts, we identified three phases of need: (1) Pre-disclosure: Offering practical, scenario-based skill training (e.g., role-playing, communication script rehearsals); (2) During disclosure: Implementing mechanisms for immediate access to professional third-party intervention (e.g., physicians, counselors); (3) Post-disclosure: Providing science education for partners, facilitating relationship mediation, and delivering legal and psychological crisis intervention for potential extreme consequences (e.g., violence, separation). These phase-specific needs emerged inductively from the interviews, not from pre-existing theoretical models. Therefore, we argue for creating a multidisciplinary collaborative support network centered on medical institutions, which should integrate infectious disease healthcare providers, psychological counselors, social workers, and legal advisors. Hospitals are encouraged to incorporate “disclosure support” into standard care protocols, establish dedicated “disclosure counseling” clinics or positions, and develop a closed-loop support pathway that includes “assessment—rehearsal—follow-up.” Simultaneously, healthcare providers should receive improved training in HIV destigmatization, effective communication techniques, and ethical considerations regarding partner disclosure—all of which directly respond to the needs voiced by our participants.

## 5 Limitations

This study has several additional limitations. First, purposive sampling was utilized, with all participants recruited from a single municipal hospital and a relatively small sample size. While qualitative studies prioritize depth over breadth, a larger and more diverse sample might have revealed additional perspectives, the generalizability of the findings necessitates validation in larger, more diverse populations (e.g., individuals from different regions, those with varied routes of infection, individuals without regular partners, or those reluctant to disclose). Future multi-site studies with larger and more diverse samples are needed to explore whether the themes identified here are consistent across broader populations of PLWH who face disclosure dilemmas. Second, as a cross-sectional interview study conducted at a single time point, it cannot capture how disclosure decisions and behaviors evolve over time, from pre-diagnosis through post-diagnosis adjustment. Future research should implement longitudinal tracking designs to examine this evolution. Future research should implement longitudinal tracking designs and develop or evaluate structured disclosure support interventions based on these findings. Third, our age criterion (18–60 years) and the exclusion of non-sexually transmitted cases limit generalizability. Future studies should specifically investigate disclosure processes in older adults living with HIV and in those infected via blood exposure or vertical transmission. Forth, our exclusion of participants with recent major negative life events (e.g., death of a loved one, relationship breakdown) or high violence risk, although ethically justified to prevent re-traumatization and ensure participant and researcher safety, may have excluded some of the most vulnerable individuals. These individuals might face the greatest challenges in HIV status disclosure to a partner. Consequently, our findings may not be generalizable to those experiencing acute life crises or at high risk of intimate partner violence. Future research should develop safe, trauma-informed methodologies to include such populations, enabling a more comprehensive understanding of disclosure dilemmas among all PLWH. Fifth, all participants in this study were recruited from a hospital setting, were virally suppressed, and voluntarily agreed to discuss their disclosure experiences. Therefore, our findings may not be transferable to individuals living with HIV who are not virally suppressed, who are not engaged in regular medical care, or who are unwilling to disclose their status to anyone. These populations may face distinct barriers (e.g., fear of transmitting HIV, lack of access to U=U evidence, greater social isolation) that were not captured in our data. Future research should specifically recruit from community settings, non-adherent populations, and those who have never disclosed, using tailored, trust-building methodologies. Sixth, despite assurances of confidentiality and the use of a non-judgmental interviewing approach, participants may have been influenced by social desirability bias - the tendency to present oneself in a favorable light. Given the sensitive nature of HIV status disclosure and the hospital recruitment context (where participants were already engaged in care), some individuals may have over-reported their motivations for disclosure (e.g., love, responsibility, moral integrity) or under-reported fears or negative experiences (e.g., selfish reasons for non-disclosure, actual discrimination encountered). Conversely, some may have exaggerated their preparatory efforts to appear competent. While we encouraged honest and open discussion, the potential for social desirability bias cannot be ruled out. Future studies should consider using anonymous self-report questionnaires or computer-assisted interviewing to supplement in-depth interviews, thereby cross-validating findings and reducing such bias.

## 6 Conclusion

Findings directly supported by participant data (within the limitations of this qualitative study: *N* = 15, single hospital, all participants virally suppressed and engaged in care): This study, based on in-depth interviews with a sample of PLWH who intended to disclose their status, systematically explores the complex psychological processes and strategic behaviors involved in their journey toward disclosure.

Within this sample, the findings indicate that the decision is shaped by a dynamic tension between internal drivers and external constraints. Although participants demonstrated considerable agency, they continued to face significant challenges, including inadequate communication skills, substantial psychological pressure, and insufficient systemic support.

Based on our data, the study offers three preliminary contributions. First, it describes the psychological process from disclosure intention to action as experienced by our participants, addressing a gap in prior research that has predominantly focused either on disclosure facilitators/barriers or on post-disclosure outcomes. Second, it identifies safety protection strategy as a recurrent theme in their narratives, suggesting that risk-averse preparation may warrants greater attention in disclosure research. Third, it proposes a tentative framework for disclosure support—assessment-rehearsal-follow-up—that could be tested in future intervention research with larger and more diverse samples.

Broader recommendations for policy, clinical practice, and future research: Based on these preliminary insights from this sample, we propose the following recommendations for consideration. These recommendations are forward-looking and should be tested and refined in larger, more diverse populations before widespread implementation: (1) in clinical practice, integrate disclosure support services into routine clinical care and establish dedicated disclosure counseling roles that engage both partners when appropriate; (2) develop multidisciplinary support networks and provide education for HIV-negative partners on U=U and supportive communication; (3) strengthen social anti-discrimination efforts and legislative protections to foster a more supportive environment for both PLWH and their partners.

## Data Availability

The original contributions presented in the study are included in the article/Supplementary material, further inquiries can be directed to the corresponding author.
