# Supplementary material for: The psychological process and support needs of HIV-positive individuals who disclose their status to sexual partners: a qualitative study
Source: Front Public Health. 2026 Jun 16;14:1870907. doi: 10.3389/fpubh.2026.1870907 (PMC13315003; doi:10.3389/fpubh.2026.1870907)
Supplement: Supplementary file 1 [file Table_1.docx]

**Supplementary data 1**

**Interview Outline**

(1) What were your primary thoughts and feelings when you decided (or considered) disclosing your infection status to your partner? What motivated you to tell him/her?

(2) During the process of making this decision, what were your biggest worries or fears?

(3) Did the attitudes of people around you toward HIV-positive individuals, or societal perceptions, influence your decision to disclose? Were you concerned that you or your family might be treated differently after disclosure?

(4) Regarding *how* to disclose, from which sources have you sought information (e.g., doctors, nurses, peers, the internet)? Do you feel the information you obtained was sufficient? What specific information or assistance do you most wish to have?

(5) In your view, what are the most critical preparations for a relatively safe and successful disclosure? (e.g., choosing the timing/location, preparing medical knowledge, psychological readiness, etc.)

(6) If you were to disclose, how would you choose the timing, location, and setting? Why do you think this approach is appropriate? Have you mentally rehearsed how to start the conversation?

(7) Are you worried that disclosure might trigger extreme reactions or violence from your partner? If so, have you considered any safety plans to protect yourself? (e.g., arranging temporary accommodation in advance, preparing ways to contact emergency services, informing trusted friends, etc.)

(8) Can you imagine what kind of reactions your partner might have (e.g., shock, anger, sadness, silence)? If the other person becomes highly emotional, even leading to arguments or conflict, what do you think you could do to protect yourself and de-escalate the situation?

(9) In your opinion, what are the most needed and practical forms of assistance that professionals such as healthcare workers or social workers in hospitals or communities could provide before, during, and after your disclosure?

(10) Looking back on our conversation today, are there any important thoughts or experiences related to "disclosing to your partner" that we haven’t discussed but that you feel are important to share?
